# Effects of a community health worker delivered intervention on maternal depressive symptoms in rural Tanzania

**DOI:** 10.1093/heapol/czaa170

**Published:** 2020-12-13

**Authors:** Lilia Bliznashka, Aisha K Yousafzai, Geofrey Asheri, Honorati Masanja, Christopher R Sudfeld

**Affiliations:** Department of Global Health and Population, Harvard T.H. Chan School of Public Health, 655 Huntington Avenue, Building 1, 11th Floor, Boston, MA 02115, USA; Department of Global Health and Population, Harvard T.H. Chan School of Public Health, 655 Huntington Avenue, Building 1, 11th Floor, Boston, MA 02115, USA; Ifakara Health Institute, Plot 463, Kiko Avenue Mikocheni, Dar es Salaam, Tanzania; Ifakara Health Institute, Plot 463, Kiko Avenue Mikocheni, Dar es Salaam, Tanzania; Department of Global Health and Population, Harvard T.H. Chan School of Public Health, 655 Huntington Avenue, Building 1, 11th Floor, Boston, MA 02115, USA; Department of Nutrition, Harvard T.H. Chan School of Public Health, 655 Huntington Avenue, Building 1, 11th Floor, Boston, MA 02115, USA

**Keywords:** Tanzania, sub-Saharan Africa, depressive symptoms, mental health, community health worker, cash transfer, responsive stimulation

## Abstract

Maternal depression affects one in four women in sub-Saharan Africa, yet evidence on effective and scalable interventions is limited. Our objective was to evaluate the effect of a community health worker (CHW) delivered home visit responsive stimulation, health and nutrition intervention, and conditional cash transfers (CCTs) for antenatal care and child growth monitoring attendance on maternal depressive symptoms. We conducted a cluster-randomized controlled trial in 12 villages in rural Ifakara, Tanzania (September 2017 to May 2019). Study villages were randomly assigned to one of three arms: (1) CHW, (2) CHW + CCT and (3) Control. Pregnant women and mothers with a child <12 months were enrolled. Maternal depressive symptoms were assessed using a Tanzanian-adapted version of the Hopkins Symptoms Checklist-25 (HSCL-25) after 18 months of follow-up. We used linear mixed-effects models to estimate intervention effects on HSCL-25 scores. Results showed that the CHW intervention significantly reduced HSCL-25 scores as compared with control [unadjusted mean difference (MD) −0.31, 95% confidence interval (CI) −0.47, −0.15]. The CHW + CCT intervention also appeared to lower HSCL-25 scores (MD −0.17, 95% CI −0.33, −0.01), but results were not statistically significant. Our findings showed that a low-intensity CHW-delivered home visit responsive stimulation, health and nutrition intervention, which did not explicitly aim to improve mental health, reduced maternal depressive symptoms, though the precise mechanisms of action remain unknown. CCTs for antenatal care and child growth monitoring appeared to provide limited to no additional benefit. Community-based integrated interventions that broadly consider maternal and child health, development and well-being have the potential to promote maternal mental health in rural Tanzania and similar settings.

**KEY MESSAGES**

- A community health worker (CHW)-delivered integrated intervention reduced maternal depressive symptoms in rural Tanzania.
- Marital status and education modified the CHW intervention effects.
- The CHW intervention plus conditional cash transfers for health visits did not appear to lower depressive symptoms.

## Introduction

Depression is a major public health concern globally (Patel *et al.*, 2018). In low- and middle-income countries (LMICs), and sub-Saharan Africa (SSA) in particular, depression is among the leading non-communicable causes of disability, affecting a higher proportion of women than men [Institute for Health Metrics and Evaluation (IHME), 2019]. In Tanzania, the setting of the present study, the percentage of disability-adjusted life years attributable to depression has nearly doubled in the past two decades [Institute for Health Metrics and Evaluation (IHME), 2019].

The prevalence of maternal perinatal common mental disorders (defined as ‘non-psychotic mental health conditions, including depression, anxiety, adjustment and somatic disorders which compromise day-to-day functioning’) in LMICs varies widely, ranging between 5% and 33% during pregnancy and between 5% and 60% after childbirth (Fisher *et al.*, 2012). In Tanzania, studies have found antenatal depression prevalence of 11.5% in the Kilimanjaro Region (Manongi *et al.*, 2020) and 30% in Dar es Salaam (Kaaya *et al.*, 2010; Rwakarema *et al.*, 2015). Likewise, studies have found postnatal depression prevalence of 12.2% in the Kilimanjaro Region (Holm-Larsen *et al.*, 2019) and 69.2% in the Singida Region (Blacker *et al.*, 2017). However, all these studies were conducted in community settings and used self-reported measures of depressive symptoms. Thus, the prevalence of clinical depression diagnosed using Diagnostic and Statistical Manual of Mental Disorders (DSM) criteria remains largely unknown in Tanzania. The major risk factors for maternal depressive symptoms established by these studies are low socioeconomic status (Rwakarema *et al.*, 2015), food insecurity (Blacker *et al.*, 2017), lack of partner support (Kaaya *et al.*, 2010; Rwakarema *et al.*, 2015), intimate partner violence, and living with HIV (Manongi *et al.*, 2020).

In LMICs, maternal perinatal depressive symptoms have been linked to pregnancy-related morbidity and mortality (Gelaye *et al.*, 2016), adverse birth outcomes (Rahman *et al.*, 2013; Accortt *et al.*, 2015; Gelaye *et al.*, 2016) and poor child health outcomes (Rahman *et al.*, 2013; Nguyen *et al.*, 2014). In some settings, maternal depressive symptoms are also associated with poor child growth (Harpham *et al.*, 2005; Surkan *et al.*, 2011; Rahman *et al.*, 2013; Nguyen *et al.*, 2014; Bennett *et al.*, 2016; Gelaye *et al.*, 2016) and development outcomes (Rahman *et al.*, 2013; Bennett *et al.*, 2016; Gelaye *et al.*, 2016). The adverse effects of maternal depressive symptoms on child health, growth and development during infancy and early childhood can persist into later childhood, adolescence and adulthood (Bennett *et al.*, 2016; Herba *et al.*, 2016).

Evidence from high-income countries suggests that the relationship between maternal depressive symptoms and child development outcomes is mediated by inadequate parenting behaviours (Goodman and Garber, 2017), such as negative affect, coercive parenting and reduced attention to child emotional expression (Dix and Meunier, 2009). Limited evidence from LMICs indicates a similar association between maternal depressive symptoms and inadequate parenting practices (Huang *et al.*, 2017), and between maternal depressive symptoms and reduced maternal sensitivity in child engagement, which is in turn associated with insecure infant attachment (Tomlinson *et al.*, 2005). However, most of this evidence is based on school-aged children and adolescents, and an evidence gap remains with respect to children younger than 2 years of age.

Parenting and responsive stimulation interventions, aimed at improving early childhood development, have been identified as a potential prevention approach to improve maternal mental health in low-resource settings (Patel *et al.*, 2018). These community-based interventions promote and protect mental health by strengthening the mother–child interaction, improving maternal mood (Rahman *et al.*, 2013) and improving parenting and caregiving practices, among other intervention-specific actions that can improve psychological well-being and create a stable environment that supports mental health (World Health Organization, 2018). However, empirical evidence on the effects of responsive stimulation interventions on maternal depressive symptoms in LMICs is mixed (Jeong *et al.*, 2018). Although a recent meta-analysis found no overall pooled effect of responsive stimulation interventions on maternal depressive symptoms (Jeong *et al.*, 2018), four of the nine studies included in the meta-analysis found significant reductions in maternal depressive symptoms in Bangladesh (Aboud *et al.*, 2013), Jamaica (Baker-Henningham *et al.*, 2005), Pakistan (Yousafzai *et al.*, 2015) and Uganda (Singla *et al.*, 2015). In addition to the nine studies included in this meta-analysis, three other studies have found positive effects of community-based responsive stimulation interventions on maternal depressive symptoms in Bangladesh (Hamadani *et al.*, 2019), Zambia (Rockers *et al.*, 2018), albeit only in unadjusted analyses, and Uganda (Atukunda *et al.*, 2019). Only four of these 12 studies were conducted in SSA countries: South Africa, Uganda (Jeong *et al.*, 2018; Atukunda *et al.*, 2019) and Zambia (Rockers *et al.*, 2018). All 12 studies varied with respect to the design and duration of the intervention, the delivery mechanism and frequency, the comparison group, the inclusion criteria, sample size and depressive symptoms assessment tool. Except for the Ugandan and Zambian interventions which specifically addressed maternal mental health (Singla *et al.*, 2015; Rockers *et al.*, 2018), none of the other 10 interventions included a psychosocial component or sought to directly improve maternal mental health. Among the studies that observed improvements in maternal depressive symptoms, authors speculated that, in addition to improved parenting competencies, one pathway through which improvements were achieved was peer support offered through regular home visits (Baker-Henningham *et al.*, 2005) and peer groups (Aboud *et al.*, 2013).

Although poverty and socioeconomic disadvantage are major risk factors for maternal depressive symptoms (Fisher *et al.*, 2012; Gelaye *et al.*, 2016), evidence on the effectiveness of poverty reduction interventions, including cash transfers, is limited and inconclusive (Lund *et al.*, 2011; Pega *et al.*, 2017; Patel *et al.*, 2018). A handful of studies have found positive effects of unconditional cash transfers on mental health among adolescents in SSA (Owusu-Addo *et al.*, 2018) and adults in South Africa (Ohrnberger *et al.*, 2020). In India, cash transfers for pregnant women conditioned on delivering in a government health facility have shown some promise in reducing maternal depressive symptoms (Powell-Jackson *et al.*, 2016). However, this evidence comes from a large social protection programme that aimed to reduce poverty rather than improve maternal mental health directly. Whether smaller conditional cash transfers (CCTs), designed to encourage clinic attendance for antenatal care (ANC) and child growth monitoring, can positively affect maternal depressive symptoms remain unclear. In addition, there is a lack of evidence on the effectiveness of CCTs when combined with responsive stimulation and nutrition interventions (Engle *et al.*, 2011). These two interventions can potentially work in synergistic ways by addressing both poverty and low parenting competencies as risk factors for maternal depression, and by linking women to health facilities for depression diagnosis and treatment.

Given the limited and mixed evidence, in the present study, we sought to evaluate the effect of an integrated home visit-based responsive stimulation, health and nutrition intervention, with and without CCTs, delivered by non-specialized trained community health workers (CHWs) on depressive symptoms among pregnant women and women with young children in rural Tanzania. The intervention was not designed to address maternal mental health directly, but we hypothesized it would reduce maternal depressive symptoms by addressing multiple risk factors and providing a range of coping strategies, e.g. strengthening mother–child interaction, improving parenting and caregiving competencies and providing peer support.

## Materials and methods

### Study design

Full details on the study design have been previously published (Sudfeld et al., 2019) . Briefly, we conducted a longitudinal cluster-randomized controlled trial (cRCT) where 12 villages stratified by urban–rural location were randomly assigned to one of three intervention arms: (1) CHW, (2) CHW + CCT and (3) Control. The CHW and CHW + CCT arms received the same integrated responsive stimulation, health and nutrition intervention. The CHW + CCT arm also received CCTs. The control arm did not receive any intervention and had access to the existing clinic-based healthcare services. In each village, enrolment in the trial continued until all pregnant women and women with a child <12 months of age were enrolled or until 50 participants were enrolled, whichever was reached first. Children with severe physical or mental impairment were excluded. The baseline survey was conducted in September to October 2017 prior to the start of the intervention; the midline survey was conducted in June to August 2018 after 9 months of intervention implementation; and the endline survey was conducted in January to May 2019 after 18 months at the completion of the intervention.

### Intervention description

Trained fieldworkers explained the intervention and associated study and obtained written informed consent from participants in their primary language. Ethical approval was received by the institutional review boards of the authors' institutions.

The intervention has been described in detail elsewhere (Sudfeld et al., 2019) . Briefly, a CHW-delivered intervention was examined alone and in combination with CCTs. Female CHWs living in the study villages’ catchment areas delivered the intervention. The intervention-specific responsive stimulation component was a Tanzanian and Swahili adapted version of the UNICEF and WHO Care for Child Development package (World Health Organization and UNICEF, 2012). This package promoted caregivers’ sensitivity and responsiveness using developmentally appropriate stimulation activities (e.g. play and communication). It consisted of essential early child development knowledge, age-appropriate play and communication activities, toy making, parenting and problem-solving counselling, and promotion of caregiver responsiveness and sensitivity. The CHWs received a 1-week classroom-based intervention-specific training prior to the start of the intervention. This training covered both theoretical and practical aspects of early child development, age-appropriate play and communication activities, counselling of caregivers, problem-solving, and making of toys and other play materials. A 3-day refresher training was conducted after 9 months of implementation, half-way through the intervention.

The health and nutrition components covered optimal antenatal, postnatal and newborn health and nutrition practices, care and danger sign identification, community-case management of childhood illness, and emergency and routine health facility referral. Training was received through a 1-year comprehensive government-certified training, which included both health and non-health-related topics. Additional health-related topics included infection and disease prevention and control, community-based reproductive health services and health promotion, home-based care, and health facility and community disease management. Non-health-related topics covered fundamentals of social work, basic life support skills, communication and customer service, computer literacy, citizenship and gender, management information systems, and fundamentals of entrepreneurship and life skills.

CHWs conducted individualized, one-on-one home visits every 4–6 weeks to participating women during which they demonstrated age-appropriate play and communication activities for the child, observed the women practicing the newly learned activities, and provided support applying the new activities. In addition, CHWs advised women on optimal health and nutrition practices for themselves and their young child, counselled them on parenting and problem-solving strategies, and made health facility referrals for emergency and routine conditions. Finally, CHWs provided counsel and advice with respect to other topics covered in the government-based training.

The CCT component consisted of monthly cash transfers of 10 000 Tanzanian shillings ($4.30) for ANC visits, with reimbursement for up to four ANC visits, or 5000 Tanzanian shillings ($2.20) for child growth monitoring and health visits, with reimbursement for up to one visit per month. Cash transfers were small relative to the $1.90 average daily per person income for smallholder farmers in Tanzania (Rapsomanikis, 2015). During the home visits, CHWs reimbursed pregnant women and women with young children after inspecting their health card or the child’s health card, respectively, to ensure the conditions for receiving the cash transfer were met. The intervention did not include a psychological component such as cognitive behavioural or behavioural activation therapy and did not explicitly address women’s mental health.

One field co-ordinator supervised the CHWs throughout the intervention. Supervision included one-on-one biweekly meetings with each CHW, a monthly meeting with all CHWs, as well as monthly home visit spot-checks where the field co-ordinator accompanied CHWs during home visits. Further details on supervision are available elsewhere (Sudfeld et al., 2019) .

### Measures

Quantitative questionnaires collected data on women’s socioeconomic and demographic characteristics, and mental health, among other data related to the primary outcomes of the trial (Sudfeld et al., 2019) . Mental health was assessed using the Hopkins Symptoms Checklist-25 (HSCL-25), which captured depression (15 questions) and anxiety (10 questions) symptoms. All questions were asked by a male fieldworker in private areas, out of sight or earshot from other household or community members. Respondents rated how much 25 anxiety- and depression-related experiences and feelings bothered or distressed them in the past 2 weeks (1 = *Not at all*, 2 = *A little*, 3 = *Quite a bit*, 4 = *Extremely).* ‘Do not know’ responses were recoded as missing. A continuous HSCL-25 score (range 1–4) was calculated as the mean of the 25 items. Higher HSCL-25 score indicated worse depressive symptoms. Separate depressive symptoms and anxiety sub-scores were also calculated as the mean of the 15 and 10 items, respectively. The HSCL-25 was previously adapted and validated for diagnosis of symptoms consistent with major depressive disorder in HIV-positive pregnant Tanzanian women (Kaaya *et al.*, 2002). In our sample, HSCL-25 revealed high internal consistency (α = 0.88) both among pregnant women and women with young children (α = 0.84 and α = 0.90, respectively). Perceived social support was assessed using the Duke University-University of North Carolina Functional Social Support Questionnaire (Broadhead *et al.*, 1988). A household wealth index was constructed using principal components analysis of 11 items assessing asset ownership and housing quality.

### Statistical analysis

Intent-to-treat intervention effects of the CHW and CHW + CCT interventions compared with control were estimated using a linear mixed-effects model. Each cluster (i.e. each village) was modelled with a different starting point and trajectory over time. Within clusters, individuals were allowed different starting points, but similar trajectories over time. Model comparison indicated that differences in individual trajectories were statistically indistinguishable. Therefore, we favoured the more parsimonious model. All models controlled for baseline values, and therefore minimized the risk of regression to the mean. Per protocol, we also estimated the effect of the pooled CHW and CHW + CCT intervention arms compared with control, as there was no indication of additional benefit provided by the CCTs. We calculated unadjusted mean differences (MDs) and 95% confidence intervals (CIs). As a measure of effect size, we calculated standardized mean differences as the unadjusted MD divided by the pooled standard deviation (SD). To assess the variability of intervention effects at the cluster level, individual HSCL-25 scores were predicted from the fitted model, and cluster level predicted HSCL-25 scores were calculated as the mean of the individual model-predicted HSCL-25 scores in each cluster. All statistical tests were two-sided. Critical values were drawn from a *t*-distribution to account for the small number of clusters (Cameron *et al.*, 2008). Results were considered statistically significant at *P *<* *0.05. Descriptive analyses were conducted in Stata Version 15 (StataCorp, 2017) and model fitting was conducted in R Version 3.6.1 (R Development Core Team, 2017).

The trial’s sample size was based on power calculations to detect meaningful changes in child development and linear growth, the co-primary outcomes (Sudfeld et al., 2019) . We conducted *post-hoc* power calculations to estimate the minimum detectable effect for the outcomes presented here. Using the same type-I error α = 0.05 and 80% power, and the sample coefficient of variation (0.05) and intra-cluster correlation (0.19 for HSCL-25, 0.17 for the depressive symptomsn sub-score and 0.15 for the anxiety sub-score), the available sample allowed us to detect effects of 0.32 SD in HSCL-25 scores, 0.39 SD in depressive symptoms sub-scores and 0.22 SD in anxiety sub-scores.

As a sensitivity analysis, we examined the potential of baseline imbalance between randomized clusters to bias estimates by including multivariate adjustment for baseline covariates. We adjusted for the following a priori selected baseline covariates: woman’s age, education (whether she had completed secondary education or not), parity (whether she was multiparous or nulliparous), marital status (whether she was married/cohabitating or not), pregnancy status at baseline (pregnant or with a child <12 months of age), woman’s perceived social support score, household wealth index and child age. In line with CONSORT guidelines, missing data on baseline covariates were imputed (Moher *et al.*, 2010) using fully conditional specifications (Li and Stuart, 2019) with 20 imputations (Graham *et al.*, 2007). In addition, we assessed possible bias due to missing HSCL-25 data due to incomplete midline and endline interviews. Baseline characteristics of women without missing HSCL-25 data at midline or endline were compared with those of women with missing HSCL-25 data using *t*-tests. First, we compared women without missing HSCL-25 data at baseline and midline to women with missing HSCL-25 data at midline. Second, we compared women without missing HSCL-25 data at baseline and endline to women with missing HSCL-25 data at endline. Differences were considered statistically significant at *P *<* *0.05. Inverse probability weights were derived from a logistic model predicting the probability of missing HSCL-25 data (i.e. probability of attrition) (Wooldridge, 2010) and applied to the effect estimates. Furthermore, we examined potential modification of the effect of the CHW and CHW + CCT interventions on HSCL-25 scores, and depressive symptoms and anxiety sub-scores by a priori defined factors: pregnancy status at baseline, maternal education, marital status, parity, symptoms consistent with depression at baseline, and number of CHW visits received (defined as having received at least 10 home visits or 90% of planned home visits, based on CHW report). To define symptoms consistent with major depressive disorder (which we refer to as depression for brevity), we used the Tanzanian cut-off of a mean score ≥1.06 on eight HSCL questions (Kaaya *et al.*, 2002) and the standard cut-off of a mean score ≥1.75 on HSCL-25 (Derogatis *et al.*, 1974). Interactions were considered statistically significant at *P *<* *0.05.

## Results

### Sample characteristics

**Figure 1:**
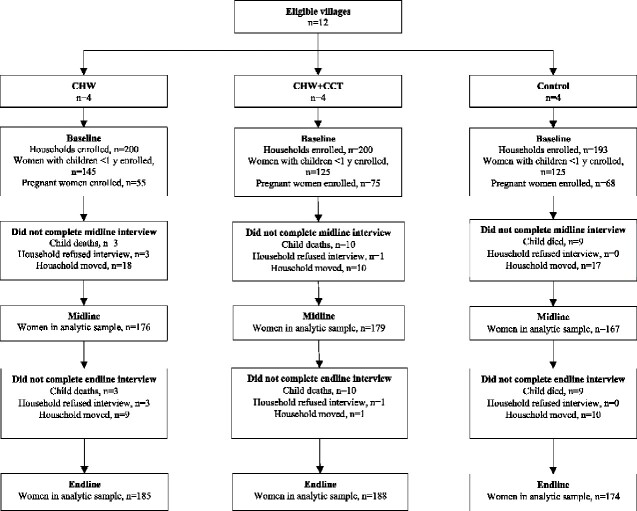
Study flow diagram. CHW, community health worker; CCT, conditional cash transfer.

A total of 593 women were enrolled in the study, 33% of whom were pregnant (Figure 1). At baseline, individual- and household-level characteristics were generally similar across intervention arms (Table 1), though there was indication of differences in parity, HSCL-25 scores and child age (Supplementary Table S1). Most women were multiparous and married or cohabitating. Education levels were low. Housing conditions were poor and access to running water limited, though most households had access to an improved latrine. HSCL-25 scores were also generally similar across intervention arms, though somewhat lower in the CHW arm. Mean HSCL-25 item scores by intervention arm and time point are shown in Supplementary Table S2.

**Table 1:** Baseline characteristics of women enrolled in the trial by intervention arm

	Control, *N* (%) or mean ± SD	CHW, *N* (%) or mean ± SD	CHW + CCT, *N* (%) or mean ± SD
Participants (*N*)	193	200	200
Woman’s characteristics			
Age (in years)	26.5 ± 6.5	27.0 ± 5.7	27.1 ± 6.7
Married or lives with partner	149 (77.2)	172 (86.0)	167 (83.5)
Completed secondary education	30 (15.5)	13 (6.5)	25 (12.5)
Is multiparous	151 (78.2)	187 (93.5)	177 (89.4)
HSCL-25 score (1–4)	1.3 ± 0.3	1.1 ± 0.2	1.3 ± 0.3
Social support score (1–4)	2.9 ± 0.9	2.1 ± 0.4	2.9 ± 0.6
Household characteristics			
Size	3.5 ± 1.9	4.0 ± 1.7	3.6 ± 2.0
Has dirt floor	86 (44.6)	85 (42.5)	125 (63.5)
Has running water	22 (11.4)	50 (25.0)	0 (0.0)
Has an improved latrine	123 (63.7)	175 (87.5)	103 (51.5)
Wealth index	0.4 ± 2.3	0.3 ± 1.7	−0.7 ± 1.6
Child characteristics			
*N*	125	145	125
Age (in months)	5.2 ± 3.6	5.3 ± 3.6	5.0 ± 3.5
Male	62 (53.7)	75 (51.7)	70 (56.0)

CHW, community health worker; CCT, conditional cash transfer; HSCL, Hopkins Symptoms Checklist.

Women with missing HSCL-25 data at midline or endline were generally similar in terms of baseline characteristics to those without missing HSCL-25 data (Supplementary Table S3A and Supplementary Table S3B). However, women without HSCL-25 data at midline were more likely to be pregnant at baseline (Supplementary Table S3A), whereas women without HSCL-25 data at endline were more likely to be nulliparous (Supplementary Table S3B). Thus, missing HSCL-25 data at midline or endline was unlikely to have a large influence on effect estimates. However, we present attrition-weighted estimates as sensitivity analyses.

### Intervention fidelity

Intervention monitoring at the CHW level showed that home visits occurred once every 4–6 weeks. After 18 months of implementation, women had received 11 CHW visits on average (25th percentile: 10 visits, and 75th percentile: 13 visits), with 71% of women receiving ≥90% of intended home visits, based on CHW report. The average CHW visit duration was 35 min in both the CHW and CHW + CCT arms. Most women recalled receiving advice on breastfeeding (88%), child health (98%), child vaccinations (82%) and responsive stimulation (95%). While home visits were intended as individualized sessions, 38% of women reported someone else was usually present during visits. Among those who reported another person was present, the majority reported that person was a friend or a neighbour (60%), another household member (21%), the child’s father/male caregiver (12%) or the mother/mother-in-law of the target woman (6%). However, nearly all women (99%) reported that fathers/male caregivers attended less than half of the CHW visits. Just over 80% of women reported discussing information received during home visits with others, a friend or a neighbour in most cases (77%). With respect to the CCT, most women (90%) reported using it to purchase clothes, toys or other goods for their child. Women reported that they alone decided how to use the CCT in nearly all cases (96%).

### Intervention effect

The effects of the CHW and CHW + CCT interventions on HSCL-25 scores, and depressive symptoms and anxiety sub-scores are presented in Table 2. The CHW intervention significantly reduced women’s HSCL-25 scores at midline by MD −0.37 (95% CI −0.49, −0.25) and at endline by MD −0.31 (95% CI −0.47, −0.15) as compared with control. Similarly, the CHW + CCT intervention reduced HSCL-25 scores by MD −0.29 (95% CI −0.42, −0.17) at midline, and MD −0.17 (95% CI −0.33, −0.01) at endline as compared with control. The CHW and CHW + CCT interventions also significantly reduced both depressive symptoms and anxiety sub-scores at midline and endline as compared with control. However, the effect of the CHW + CCT intervention on the anxiety sub-score at endline did not reach statistical significance. Reductions in sub-scores for depressive symptoms appeared larger than reductions in sub-scores for anxiety. The sensitivity analyses adjusting for confounders and accounting for missing HSCL-25 data (Table 2 and Supplementary Table S4, respectively) showed overall similar intervention effects at both midline and endline. Assuming no interaction between the CHW and CCT interventions, the pooled intervention arms (CHW and CHW + CCT combined) significantly reduced HSCL-25 scores, and depressive symptoms and anxiety sub-scores as compared with control at both midline and endline (Supplementary Table S5).

**Table 2:** Effect of the CHW and CHW + CCT interventions on women’s HSCL-25 score, and depressive symptoms and anxiety sub-scores at midline (9 months) and endline (18 months)

				Unadjusted		Multivariate adjusted^a^	
	Control, mean ± SD	CHW, mean ± SD	CHW + CCT, mean ± SD	CHW vs control, mean difference (95% CI)	CHW + CCT vs control, mean difference (95% CI)	CHW vs control, mean difference (95% CI)	CHW + CCT vs control, mean difference (95% CI)
Participants (*N*)	193	200	200				
Mean HSCL-25 score							
Baseline	1.26 ± 0.31	1.13 ± 0.20	1.34 ± 0.32				
Midline	1.41 ± 0.39	1.04 ± 0.06	1.12 ± 0.20	−0.37	−0.29	−0.43	−0.29
				(−0.49, −0.25)**	(−0.42, −0.17)**	(−0.52, −0.33)**	(−0.39, −0.20)**
Endline	1.34 ± 0.35	1.04 ± 0.10	1.18 ± 0.23	−0.31	−0.17	−0.36	−0.17
				(−0.47, −0.15)**	(−0.33, −0.01)	(−0.50, −0.23)**	(−0.30, −0.04)*
Mean depressive symptoms sub-score							
Baseline	1.30 ± 0.39	1.10 ± 0.25	1.37 ± 0.37				
Midline	1.46 ± 0.46	1.05 ± 0.09	1.15 ± 0.27	−0.41	−0.32	−0.49	−0.32
				(−0.54, −0.28)**	(−0.45, −0.19)**	(−0.59, −0.38)**	(−0.42, −0.22)**
Endline	1.40 ± 0.43	1.04 ± 0.13	1.12 ± 0.19	−0.36	−0.20	−0.43	−0.19
				(−0.52, −0.21)**	(−0.35, −0.04)*	(−0.56, −0.30)**	(−0.32, −0.07)*
Mean anxiety sub-score							
Baseline	1.20 ± 0.23	1.16 ± 0.18	1.29 ± 0.33				
Midline	1.33 ± 0.37	1.03 ± 0.06	1.09 ± 0.16	−0.31	−0.25	−0.33	−0.26
				(−0.43, −0.19)**	(−0.38, −0.13)**	(−0.44, −0.22)**	(−0.36, −0.15)**
Endline	1.25 ± 0.33	1.03 ± 0.09	1.12 ± 0.19	−0.23	−0.13	−0.25	−0.14
				(−0.41, −0.05)*	(−0.31, 0.04)	(−0.42, −0.09)*	(−0.30, 0.03)

^a^ Effect estimates controlled for the following baseline covariates: women’s age, pregnancy status at enrolment, education, parity, marital status, perceived social support, household wealth and child age.

CHW, community health worker; CCT, conditional cash transfer; HSCL, Hopkins Symptoms Checklist.

*P < 0.05, **P < 0.01.

**Figure 2:**
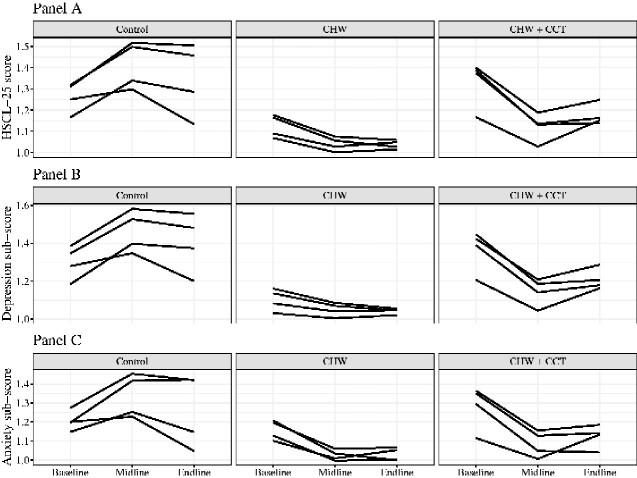
Between-cluster within intervention arm variability in predicted Hopkins Symptoms Checklist-25 (HSCL-25) scores (Panel A), predicted depression sub-scores (Panel B), and predicted anxiety sub-scores (Panel C). Lines represent mean cluster-level predicted HSCL-25 scores from an unadjusted linear mixed effects model. Possible range for each score is 1 to 4. Abbreviations used: CHW, community health worker; CCT, conditional cash transfer; HSCL, Hopkins Symptoms Checklist..

The variability of intervention effects at the cluster level is shown in Figure 2. Despite some variability in mean cluster-level HSCL-25 scores at baseline, changes over time were generally similar across clusters within the same intervention arm, though variability in slopes seemed to increase over time with somewhat larger differences in slopes between midline and endline than between baseline and midline (Figure 2A). All CHW clusters had lower predicted mean cluster-level HSCL-25 scores compared with control clusters at both midline and endline. Similarly, all CHW + CCT clusters had lower predicted mean scores compared with control clusters at midline, and all but one of the control clusters at endline. Cluster-level variability of intervention effects on the depressive symptoms and anxiety sub-scores was generally similar to results for the full HSCL-25 score (Figure 2B and C).

In addition, we found several potential modifiers of the effect of the interventions on HSCL-25 scores. At midline, the effects of the CHW and CHW + CCT interventions were greater among women without secondary education (*P*-values for interaction <0.05, Supplementary Table S6A). At endline, CHW and CHW + CCT intervention effects were greater among married or cohabitating women as compared with single women. Furthermore, CHW + CCT intervention effects were greater among women without symptoms consistent with depression at baseline (based on both the Tanzanian and US cut-offs) as compared with women with symptoms consistent with depression (*P-*values for interaction <0.05, Supplementary Table S6B). There was no evidence of interaction (all *P*-values for interaction >0.10) between either the CHW or CHW + CCT interventions and pregnancy status at baseline or parity on HSCL-25 scores at midline or endline (Supplementary Table S6A and Supplemental Table S6B, respectively). Results on potential effect modifiers of the effects of the CHW and CHW + CCT interventions on the depressive symptoms and anxiety sub-scores were overall similar to those for HSCL-25 (Supplementary Table 6A and Supplemental Table S6B). Importantly, reductions in depression sub-scores were larger among women without symptoms consistent with depression at baseline in both the CHW and CHW + CCT intervention arms. Lastly, the CHW and CHW + CCT intervention effects on HSCL-25, depressive symptoms and anxiety sub-scores appeared similar regardless of whether women completed ≥90% or <90% of home visits (Supplementary Table S7).

## Discussion

In this cRCT conducted in rural Tanzania, both the CHW and CHW + CCT interventions reduced maternal depressive symptoms as compared with control. However, there was little to no evidence that the CCTs tied to antenatal care and child growth monitoring attendance provided additional benefit to the CHW intervention. After 18 months, the reductions in depressive symptoms in the CHW intervention arm corresponded to an effect size of −1.20. However, the wide confidence intervals indicated that moderate to very large reductions were possible, with effect sizes ranging from −0.58 to −1.83. Similarly, the CHW + CCT intervention may have reduced depressive symptoms (effect sizes ranged from −0.04 to −1.11), but these results did not reach statistical significance. Larger reductions were observed in depressive symptoms sub-scores as compared with anxiety sub-scores. In addition, we found that some subgroups of women appeared to benefit more from the intervention, specifically women who were married or cohabitating, and women with less than secondary education. Importantly, although the intervention reduced depressive symptoms among women with symptoms consistent with depression at baseline, reductions were greater among women without symptoms consistent with depression at baseline.

This responsive stimulation, health and nutrition intervention was delivered by CHWs who completed a 1-year comprehensive government-certified health- and non-health-related training. While none of the individual intervention or training components specifically aimed to promote maternal mental health, the holistic nature of the package addressed several underlying risk factors for depressive symptoms. The CHW core curriculum intended to support maternal and child health and nutrition, and build and provide social support, whereas the additional responsive stimulation component aimed to improve parenting competencies. The entrepreneurship and life skills training of the CHWs potentially addressed economic risk factors, though our data did not allow us to assess the extent to which this occurred. Nevertheless, the holistic intervention package provided a variety of coping strategies (e.g. social support, life skills, parenting competencies) particularly to women who were more exposed to risk factors (i.e. those with low education and those who were married/cohabitating and possibly experienced more relationship stressors) and who were therefore more likely at higher risk of depressive symptoms. The larger intervention effects among women without depressive symptoms at baseline also suggest that the intervention was a successful strategy to promote women’s mental health broadly by addressing multiple risk factors (e.g. low parenting competencies, lack of partner and peer support) and providing a variety of coping strategies [e.g. improved parenting and caregiving knowledge and skills, improved maternal responsiveness and sensitivity towards the infant, improved problem-solving skills (Rahman *et al.*, 2013)]. Notably, the intervention did not appear to be as effective in reducing depressive symptoms. Given the integrated nature of the intervention package and our study design, we could not disentangle which intervention components contributed to the large improvements in maternal mental health we found. However, the multiple components likely had cumulative and synergistic effects by addressing multiple rather than individual risk factors for depressive symptoms. Future studies should be carefully designed to unpack the mechanisms through which complex integrated interventions work to achieve impact, and the cumulative and synergistic effects of delivering multiple intervention components.

Compared with other responsive stimulation interventions, with or without a nutrition component, that have previously been shown to improve maternal mental health in LMICs (Jeong *et al.*, 2018; Rockers *et al.*, 2018; Atukunda *et al.*, 2019; Hamadani *et al.*, 2019), the effect sizes we found were much larger. In Pakistan, a similar intervention integrating responsive stimulation (using the UNICEF-WHO Care for Child Development package) and enhanced nutrition, delivered through monthly CHW visits for 24 months, only found modest effects (−0.2 SD) on maternal mental health (Yousafzai *et al.*, 2015). The study provided an estimate of the added benefit of the integrated intervention package compared with standard home visits (Yousafzai *et al.*, 2015) rather than compared with no home visits (as we did in the present study). Therefore, we expected the intervention effects we isolated to be larger than those in the Pakistan study. In addition, although longer, the training received by the CHWs in Pakistan did not include non-health-related topics (Yousafzai *et al.*, 2014) similar to the ones in the Tanzanian government curriculum. Addressing other risk factors, in addition to low parenting competencies, could have contributed to the larger improvements in maternal depressive symptoms we observed. Together with other study- and context-related aspects, these differences can help explain the differences in effect sizes between the two studies. Of note is that the range of effect sizes we found was about 1.5–4.7 times larger than the −0.39 SD effect size of an integrated responsive stimulation and nutrition intervention that explicitly aimed to improve maternal psychological well-being through mother-care sessions in Uganda (Singla *et al.*, 2015). However, the many differences between the Ugandan study and ours, including the delivery model, duration and target population, render our results difficult to compare.

Similar to prior studies, we measured self-reported depressive symptoms not clinical depression. The role of the CHW is likely to be promotive of good mental health in women, enabling coping with daily life stressors and reducing risks for maternal depressive symptoms. While this intervention appears to be a promising strategy for promoting and protecting mental health even in populations and individuals with relatively low depressive symptomology, our data also support the need for supplemental interventions and strategies to diagnose and treat women with clinical depression by trained mental health professionals. One potential area of future research is assessing the acceptability, feasibility, and effectiveness of tying monetary incentives, such as CCTs, to a mental health screening assessment by a trained medical professional.

Among the studies examining the effect of integrated responsive stimulation, health and nutrition interventions on maternal mental health in LMICs, this is the first study to examine the variability of impacts. Despite some variability between clusters at the start of the intervention, reductions over time were similar within intervention arms. Although differences between cluster means were small at baseline, individual item means differed across intervention arms indicating that different symptoms likely contributed to overall depressive symptomology in each arm. These results suggest that the CHW intervention was likely successful in reducing depressive symptoms regardless of their initial severity and highlight the potential of home visit-based integrated interventions to address a variety of depressive symptomatology.

Although we did not have a CCT only arm, we were able to qualitatively assess whether the intervention worked to improve mental health through the responsive stimulation, health and nutrition component alone (CHW arm) or through the combination of the latter component with CCTs and additional contacts with the health system conditioned by the CCT (CHW + CCT arm). Our findings indicated similar reductions in depressive symptoms in both the CHW and CHW + CCT intervention arms, suggesting that the intervention effects were likely due to the responsive stimulation, health and nutrition component rather than the CCT component. While maternal perinatal visits and child growth monitoring visits may be beneficial for both mothers and children, these did not seem to have played an additional role in reducing depressive symptoms. These findings suggest that in this setting, home visit-based interventions may be sufficient to help improve maternal depressive symptoms. Alternatively, it is possible that the CCT amount (which covered travel expenses to the health facility) was sufficient to promote adherence to child growth monitoring and health visits, but insufficient to adequately address the economic risk factors for maternal depressive symptoms in this setting. Future studies should test the effectiveness of larger CCTs on improving maternal mental health. Finally, the somewhat smaller improvements in maternal depressive symptoms in the CHW + CCT arm relative to the CHW arm could be explained by potential adverse effect of CCTs, e.g. shortened home visit duration due to the formalities of the CCT receipt or the CCTs occupying maternal attention and focus. These finding may also be the result of chance.

Furthermore, our study did not vary intervention duration, i.e. 9 vs 18 months. Therefore, it remains unclear whether the large reductions in maternal depressive symptoms observed after 9 months of intervention implementation would have been sustained at endline in the absence of home visits. Shorter interventions, ranging from 6 to 12 months, have previously demonstrated positive effects on maternal depressive symptoms (Baker-Henningham *et al.*, 2005; Aboud *et al.*, 2013; Singla *et al.*, 2015). Shorter interventions will likely be more cost-effective reaching more women at the same cost as longer interventions delivered to fewer women. Future studies should assess the optimal duration of home visit interventions to achieve improvements in maternal depressive symptoms. In addition, we assessed intervention effects shortly after the end of the 18-month intervention, and therefore cannot elucidate long-term post-intervention effects. Future work should assess the long-term sustainability of intervention effects, particularly relative to intervention duration, to help inform the design of successful, cost-effective interventions to sustainably improve maternal depressive symptoms.

The intervention was assessed through a rigorous cRCT that was closely monitored and demonstrated high fidelity. However, our study is not without limitations. First, while the HSCL-25 scale has been previously validated for use among HIV-positive pregnant women in urban Tanzania (Kaaya *et al.*, 2002), it has not been validated for use among pregnant women and women with young children with unknown HIV status living in rural areas. Nevertheless, the HSCL-25 scale showed high internal consistency in our sample, as well as separately among pregnant and non-pregnant women. In addition, the depressive symptoms sub-score of the HSCL has been validated for use among other SSA populations, including mothers with young children in the Democratic Republic of the Congo (Bass *et al.*, 2008), and the general population in Rwanda (Bolton, 2001) and Lesotho (Hollifield *et al.*, 1990). Second, missing HSCL-25 data at midline and endline reduced the size of the analytic sample. However, results presented earlier showed few differences between women without missing HSCL-25 data and those with missing HSCL-25 data. The magnitude and significance of the intervention effects changed little after adjusting for potential bias due to missing HSCL-25 data. Third, unmeasured confounders, such as HIV status and experience of intimate partner violence which are associated with women’s depressive symptoms in Tanzania (Manongi *et al.*, 2020), could have potentially lowered women’s ability to participate in and benefit from the intervention. If this were the case, we may be overestimating intervention effects, though it is unclear how such confounders would have interacted or been influenced by the intervention. Finally, our study was conducted in a small number of villages. A single village doing exceptionally well or exceptionally poorly in terms of depressive symptoms could have a larger influence and skew our effect estimates. However, our assessment of the variability of intervention effects showed that all CHW and CHW + CCT villages had overall similar trajectories of depressive symptoms over time and it is likely that no single village skewed our results. Nevertheless, given the small number of rural villages, our results may not be generalizable to all settings. Future studies should replicate our findings at a larger scale in Tanzania, as well as in diverse settings in SSA and other LMICs.

## Conclusion

In conclusion, this low-intensity home visit-based, integrated, CHW-delivered responsive stimulation, health and nutrition intervention targeting pregnant women and women with young children in rural Tanzania significantly reduced maternal depressive symptoms. We observed larger improvements among women at potentially higher risk of depression, i.e. those who were married or cohabitating, and those with less than secondary education. The larger intervention effects among women without depressive symptoms at baseline indicate the intervention is a promising strategy to improve and promote broader mental health. Improved peer support, parenting competencies, and problem-solving strategies were likely the mechanisms through which the intervention worked, though our data and the intervention design did not permit us to formally explore these mechanisms. However, the intervention did not appear sufficient to meet the needs of women with symptoms of clinical depression. Our findings have important implications for policy and practice. First, responsive stimulation, health and nutrition interventions can successfully support women at risk of depression potentially through targeting a range of risks and fostering multiple coping strategies. Given the large treatment gaps, lack of access to quality care globally and limited financial resources allocated to mental health in LMICs (Patel *et al.*, 2018), these types of integrated interventions can help promote and protect mental health while health systems build the financial and human resources capacity to tackle mental health challenges. Second, our findings suggested little to no additional benefit of CCTs when combined with the CHW intervention. More work is needed to understand the optimal size and conditionality of monetary incentives before they can be successfully used to address maternal depressive symptoms in this and similar types of low-resource settings. Finally, given the intensive and lengthy CHW training, future research should assess the feasibility and cost-effectiveness of building a large CHW workforce and scaling-up this intervention.

## Supplementary data

Supplementary data are available at *Health Policy and Planning* online.

*Conflict of interest statement.* None declared.

## Funding

This work was supported by Grand Challenges Canada [grant number #R-SBPOC-1707-09024]. The funder had no role in the study design; collection, analysis and interpretation of the data; writing of the results; or in the decision to submit the article for publication.

*Ethical approval.* Ethical approval was received by the institutional review boards of the Harvard T.H. Chan School of Public Health (reference number IRB17-1001) and the Ifakara Health Institute in Tanzania (reference number 007-2017), and the National Health Research Ethics Sub-Committee of the Tanzanian National Institute of Medical Research (reference number NIMR/HQ/R.8a/Vol.IX/2538). The trial was registered with *ISRCTN registry* as ISRCTN10323949.

## Supplementary Material

czaa170_Supp
