# Supplementary material for: Effects of a community health worker delivered intervention on maternal depressive symptoms in rural Tanzania
Source: Health Policy Plan. 2020 Dec 13;36(4):473–83. doi: 10.1093/heapol/czaa170 (PMC8128007; doi:10.1093/heapol/czaa170)
Supplement: czaa170_Supp [file czaa170_supp.zip › R1. Supplementary Data.docx]

**Table 1** Assessment of potential differences in baseline characteristics of women enrolled in the trial by intervention arm^a^

|  | Control  N (%) or  Mean ± SD | CHW  N (%) or  Mean ± SD | CHW+CCT  N (%) or  Mean ± SD | p-value |
| --- | --- | --- | --- | --- |
| Participants (N) | 193 | 200 | 200 |  |
| Woman’s characteristics |  |  |  |  |
| Age (in years) | 26.5 ± 6.5 | 27.0 ± 5.7 | 27.1 ± 6.7 | 0.200 |
| Married or lives with partner | 149 (77.2) | 172 (86.0) | 167 (83.5) | 0.287 |
| Completed secondary education | 30 (15.5) | 13 (6.5) | 25 (12.5) | 0.119 |
| Is multiparous | 151 (78.2) | 187 (93.5) | 177 (89.4) | 0.034 |
| HSCL-25 score (1-4) | 1.3±0.3 | 1.1±0.2 | 1.3±0.3 | 0.054 |
| Social support score (1-4) | 2.9 ± 0.9 | 2.1 ± 0.4 | 2.9 ± 0.6 | 0.143 |
| Household characteristics |  |  |  |  |
| Size | 3.5±1.9 | 4.0±1.7 | 3.6±2.0 | 0.369 |
| Has dirt floor | 86 (44.6) | 85 (42.5) | 125 (63.5) | 0.869 |
| Has running water | 22 (11.4) | 50 (25.0) | 0 (0.0) | 0.316 |
| Has an improved latrine | 123 (63.7) | 175 (87.5) | 103 (51.5) | 0.188 |
| Wealth index | 0.4 ± 2.3 | 0.3 ± 1.7 | -0.7 ± 1.6 | 0.929 |
| Child characteristics |  |  |  |  |
| N | 125 | 145 | 125 |  |
| Age (in months) | 5.2±3.6 | 5.3±3.6 | 5.0±3.5 | 0.097 |
| Male | 62 (53.7) | 75 (51.7) | 70 (56.0) | 0.744 |

^a^ Abbreviations used: CHW, community health worker; CCT, conditional cash transfer; HSCL, Hopkins Symptoms Checklist.

**Supplementary Tables 2** Hopkins systems checklist-25 (HSCL-25) item scores at each time point by intervention arm^a^

|  |  | Control  Mean ± SD | CHW  Mean ± SD | CHW+CCT  Mean ± SD |
| --- | --- | --- | --- | --- |
| Participants (N) |  | 193 | 200 | 200 |
| Feeling low in energy, slowed down | Baseline | 1.8±1.0 | 1.2±0.5 | 1.8±0.8 |
|  | Midline | 1.9±1.0 | 1.2±0.4 | 1.6±0.6 |
|  | Endline | 1.5±0.7 | 1.2±0.4 | 1.5±0.6 |
| Blaming self for things | Baseline | 1.4±0.8 | 1.2±0.5 | 1.4±0.7 |
|  | Midline | 1.8±0.9 | 1.3±0.5 | 1.1±0.4 |
|  | Endline | 1.6±0.7 | 1.1±0.3 | 1.3±0.7 |
| Crying easily | Baseline | 1.2±0.5 | 1.1±0.4 | 1.3±0.6 |
|  | Midline | 1.4±0.8 | 1.0±0.1 | 1.0±0.3 |
|  | Endline | 1.2±0.4 | 1.0±0.1 | 1.2±0.6 |
| Loss of sexual interest or pleasure | Baseline | 1.8±1.2 | 1.1±0.4 | 1.7±0.9 |
|  | Midline | 2.0±1.1 | 1.0±0.2 | 1.3±0.6 |
|  | Endline | 2.1±1.0 | 1.1±0.3 | 1.4±0.8 |
| Poor appetite | Baseline | 1.3±0.7 | 1.1±0.5 | 1.4±0.7 |
|  | Midline | 1.4±0.8 | 1.1±0.2 | 1.1±0.4 |
|  | Endline | 1.3±0.7 | 1.0±0.2 | 1.2±0.5 |
| Difficulty falling asleep or staying asleep | Baseline | 1.1±0.4 | 1.2±0.5 | 1.4±0.8 |
|  | Midline | 1.4±0.7 | 1.1±0.2 | 1.1±0.4 |
|  | Endline | 1.3±0.7 | 1.1±0.3 | 1.1±0.4 |
| Feeling hopeless about the future | Baseline | 1.1±0.4 | 1.1±0.4 | 1.2±0.5 |
|  | Midline | 1.2±0.7 | 1.0±0.1 | 1.1±0.4 |
|  | Endline | 1.2±0.5 | 1.0±0.2 | 1.1±0.4 |
| Feeling sad or blue | Baseline | 1.3±0.6 | 1.1±0.4 | 1.4±0.7 |
|  | Midline | 1.4±0.7 | 1.0±0.2 | 1.2±0.5 |
|  | Endline | 1.4±0.7 | 1.0±0.2 | 1.2±0.5 |
| Feeling lonely | Baseline | 1.1±0.4 | 1.1±0.5 | 1.4±0.7 |
|  | Midline | 1.3±0.7 | 1.0±0.1 | 1.2±0.5 |
|  | Endline | 1.2±0.6 | 1.0±0.2 | 1.2±0.5 |
| Thoughts of ending one’s life | Baseline | 1.0±0.1 | 1.0±0.1 | 1.1±0.4 |
|  | Midline | 1.1±0.5 | 1.0±0.1 | 1.0±0.3 |
|  | Endline | 1.0±0.3 | 1.0±0.1 | 1.0±0.3 |
| Feeling trapped or caught | Baseline | 1.1±0.4 | 1.0±0.2 | 1.1±0.5 |
|  | Midline | 1.2±0.5 | 1.0±0.1 | 1.1±0.3 |
|  | Endline | 1.2±0.6 | 1.0±0.2 | 1.1±0.3 |
| Worrying too much about things | Baseline | 1.2±0.6 | 1.1±0.3 | 1.4±0.8 |
|  | Midline | 1.4±0.7 | 1.0±0.1 | 1.2±0.5 |
|  | Endline | 1.5±0.8 | 1.0±0.1 | 1.2±0.5 |
| Feeling no interest in things | Baseline | 1.2±0.6 | 1.1±0.3 | 1.3±0.6 |
|  | Midline | 1.6±0.7 | 1.0±0.1 | 1.1±0.3 |
|  | Endline | 1.5±0.7 | 1.0±0.1 | 1.1±0.4 |
| Feeling everything is an effort | Baseline | 1.7±1.1 | 1.0±0.3 | 1.5±0.7 |
|  | Midline | 1.7±0.8 | 1.0±0.1 | 1.1±0.4 |
|  | Endline | 1.6±0.8 | 1.0±0.1 | 1.3±0.5 |
| Feeling worthless | Baseline | 1.1±0.4 | 1.1±0.3 | 1.3±0.7 |
|  | Midline | 1.3±0.7 | 1.0±0.1 | 1.1±0.4 |
|  | Endline | 1.2±0.6 | 1.0±0.1 | 1.1±0.4 |
| Feeling suddenly scared or afraid for no reason | Baseline | 1.1±0.4 | 1.0±0.2 | 1.3±0.6 |
|  | Midline | 1.3±0.5 | 1.0±0.1 | 1.1±0.3 |
|  | Endline | 1.1±0.4 | 1.0±0.1 | 1.1±0.2 |
| Feeling fear | Baseline | 1.1±0.3 | 1.0±0.2 | 1.3±0.6 |
|  | Midline | 1.3±0.6 | 1.0±0.1 | 1.0±0.2 |
|  | Endline | 1.1±0.5 | 1.0±0.1 | 1.1±0.2 |
| Faintness, dizziness, weakness | Baseline | 1.3±0.5 | 1.2±0.5 | 1.3±0.7 |
|  | Midline | 1.2±0.7 | 1.0±0.2 | 1.1±0.3 |
|  | Endline | 1.2±0.6 | 1.0±0.2 | 1.1±0.4 |
| Nervousness or shakiness inside | Baseline | 1.1±0.4 | 1.1±0.3 | 1.2±0.5 |
|  | Midline | 1.3±0.5 | 1.0±0.0 | 1.0±0.1 |
|  | Endline | 1.1±0.4 | 1.0±0.2 | 1.0±0.2 |
| Heart pounding or racing | Baseline | 1.5±0.7 | 1.4±0.6 | 1.7±0.9 |
|  | Midline | 1.5±0.9 | 1.1±0.3 | 1.2±0.5 |
|  | Endline | 1.5±0.8 | 1.1±0.3 | 1.3±0.6 |
| Trembling | Baseline | 1.0±0.2 | 1.0±0.2 | 1.1±0.5 |
|  | Midline | 1.1±0.3 | 1.0±0.1 | 1.1±0.3 |
|  | Endline | 1.1±0.3 | 1.0±0.1 | 1.1±0.3 |
| Feeling tense or keyed up | Baseline | 1.2±0.5 | 1.1±0.4 | 1.2±0.6 |
|  | Midline | 1.4±0.8 | 1.0±0.1 | 1.0±0.2 |
|  | Endline | 1.4±0.7 | 1.0±0.1 | 1.1±0.3 |
| Headaches | Baseline | 1.6±0.5 | 1.4±0.6 | 1.6±0.9 |
|  | Midline | 2.0±1.1 | 1.1±0.3 | 1.3±0.5 |
|  | Endline | 1.8±0.8 | 1.1±0.3 | 1.5±0.7 |
| Spells of terror, intense fear, or panic | Baseline | 1.0±0.1 | 1.0±0.2 | 1.1±0.4 |
|  | Midline | 1.2±0.5 | 1.0±0.0 | 1.0±0.2 |
|  | Endline | 1.1±0.3 | 1.0±0.1 | 1.0±0.2 |
| Feeling restless, can't sit still | Baseline | 1.1±0.4 | 1.3±0.5 | 1.1±0.3 |
|  | Midline | 1.0±0.2 | 1.0±0.1 | 1.0±0.2 |
|  | Endline | 1.0±0.3 | 1.0±0.0 | 1.0±0.1 |

^a^ Each item is scored on a 4-point scale: 1 = ‘‘not at all’’, 2 = ‘‘a little’’, 3 = ‘‘quite a bit’’, and 4 = ‘‘extremely’’. Abbreviations used: CHW, community health worker; CCT, conditional cash transfer; HSCL, Hopkins Symptoms Checklist.

**Supplementary Table 3A** Baseline characteristics of women who were interviewed at baseline and midline and women who were interviewed only at baseline^a^

|  | Completed midline interview  N (%) or Mean ± SD | Did not complete midline interview  N (%) or Mean ± SD | p-value^b^ |
| --- | --- | --- | --- |
| Participants (N) | 522 | 71 |  |
| Woman’s characteristics |  |  |  |
| Married or living with partner | 436 (83.5) | 52 (73.2) | 0.10 |
| Completed secondary education | 62 (11.9) | 6 (8.5) | 0.43 |
| Pregnant at baseline | 164 (31.4) | 33 (46.5) | 0.04 |
| Is multiparo­us | 459 (88.1) | 56 (80.0) | 0.05 |
| Hopkins Symptoms Checklist-25 (HSCL-25) Score | 1.2±0.3 | 1.2±0.2 | 0.02 |
| Social support score (1-4) | 2.6±0.8 | 2.7±0.7 | 0.53 |
| Household characteristics |  |  |  |
| Size | 3.7±1.8 | 3.8±2.0 | 0.65 |
| Has dirt floor | 258 (49.7) | 38 (53.5) | 0.53 |
| Has running water | 62 (11.9) | 10 (14.1) | 0.56 |
| Has an improved latrine | 351 (67.2) | 50 (70.4) | 0.55 |
| Wealth index | 0.0±1.9 | -0.2±1.8 | 0.29 |

^a^ Abbreviations used: CHW, community health worker; CCT, conditional cash transfer; HSCL, Hopkins Symptoms Checklist.

^b^ Based on a t-test comparing women interviewed at baseline and midline and women interviewed at baseline only.

**Supplementary Table 3B** Baseline characteristics of women who were interviewed at baseline and endline and women who were interviewed only at baseline^a^

|  | Completed endline interview  N (%) or Mean ± SD | Did not complete endline interview  N (%) or Mean ± SD | p-value^b^ |
| --- | --- | --- | --- |
| Participants (N) | 547 | 46 |  |
| Woman’s characteristics |  |  |  |
| Married or living with partner | 453 (82.8) | 35 (76.1) | 0.35 |
| Completed secondary education | 62 (11.3) | 6 (13.0) | 0.78 |
| Pregnant at baseline | 172 (31.4) | 25 (54.3) | 0.06 |
| Is multiparous | 480 (87.9) | 35 (77.8) | 0.05 |
| Mean Hopkins Symptoms Checklist-25 (HSCL-25) Score | 1.2±0.3 | 1.2±0.2 | 0.06 |
| Social support score (1-4) | 2.6±0.8 | 2.7±0.8 | 0.88 |
| Household characteristics |  |  |  |
| Size | 3.7±1.9 | 3.5±1.9 | 0.55 |
| Has dirt floor | 274 (50.4) | 22 (47.8) | 0.72 |
| Has running water | 66 (12.1) | 6 (13.0) | 0.81 |
| Has an improved latrine | 369 (67.5) | 32 (69.6) | 0.62 |
| Wealth index | 0.0±1.9 | -0.2±1.8 | 0.37 |

^a^ Abbreviations used: CHW, community health worker; CCT, conditional cash transfer; HSCL, Hopkins Symptoms Checklist.

^b^ Based on a t-test comparing women interviewed at baseline and midline and women interviewed at baseline only.

**Supplemental Table 4** Attrition-weighted effects of the CHW and CHW+CCT interventions on women’s Hopkin’s symptoms checklist-25 (HSCL-25) score, and depression and anxiety sub-scores at midline (9 months) and endline (18 months)^a^

|  | Control  Mean ± SD | CHW  Mean ± SD | CHW vs. Control  Mean difference  (95% CI) | CHW+CCT  Mean ± SD | CHW+CCT vs. Control  Mean difference  (95% CI) |
| --- | --- | --- | --- | --- | --- |
| Participants (N) | 193 | 200 |  | 200 |  |
| **Mean HSCL-25 score** |  |  |  |  |  |
| Baseline | 1.26 ± 0.31 | 1.13 ± 0.20 |  | 1.34 ± 0.32 |  |
| Midline | 1.41 ± 0.39 | 1.04 ± 0.06 | -0.34 (-0.47, -0.21)** | 1.12 ± 0.20 | -0.25 (-0.38, -0.12)** |
| Endline | 1.34 ± 0.35 | 1.04 ± 0.10 | -0.25 (-0.47, -0.03)* | 1.18 ± 0.23 | -0.11 (-0.33, 0.11) |
| **Mean depression sub-score** |  |  |  |  |  |
| Baseline | 1.30 ± 0.39 | 1.10 ± 0.25 |  | 1.37 ± 0.37 |  |
| Midline | 1.46 ± 0.46 | 1.05 ± 0.09 | -0.39 (-0.55, -0.24)** | 1.15 ± 0.27 | -0.27 (-0.42, -0.12)** |
| Endline | 1.40 ± 0.43 | 1.04 ± 0.13 | -0.29 (-0.53, -0.06)* | 1.12 ± 0.19 | -0.12 (-0.35, 0.12) |
| **Mean anxiety sub-score** |  |  |  |  |  |
| Baseline | 1.20 ± 0.23 | 1.16 ± 0.18 |  | 1.29 ± 0.33 |  |
| Midline | 1.33 ± 0.37 | 1.03 ± 0.06 | -0.29 (-0.42, -0.16)** | 1.09 ± 0.16 | -0.24 (-0.37, -0.11)** |
| Endline | 1.25 ± 0.33 | 1.03 ± 0.09 | -0.19 (-0.43, 0.04) | 1.12 ± 0.19 | -0.10 (-0.34, 0.13) |

^a^ Abbreviations used: CHW, community health worker; CCT, conditional cash transfer; HSCL, Hopkins Symptoms Checklist. Statistical significance: * p<0.05, ** p<0.01.

**Supplemental Table 5** Effect of the pooled CHW and CHW+CCT intervention arms on women’s Hopkin’s symptoms checklist-25 (HSCL-25) score, and depression and anxiety sub-scale scores at midline (9 months) and endline (18 months)^a^

|  | Control  N (%) or  Mean ± SD | Intervention arms  (CHW or CHW+CCT)  N (%) or  Mean ± SD | Intervention arms vs. Control  Mean difference or Relative risk (95% CI) |
| --- | --- | --- | --- |
| Participants (N) | 193 | 400 |  |
| **Mean HSCL-25 score** |  |  |  |
| Baseline | 1.26 ± 0.31 | 1.23 ± 0.28 |  |
| Midline | 1.41 ± 0.39 | 1.08 ± 0.15 | -0.33 (-0.46, -0.20)** |
| Endline | 1.34 ± 0.35 | 1.11 ± 0.19 | -0.24 (-0.38, -0.10)** |
| **Mean depression sub-score** |  |  |  |
| Baseline | 1.30 ± 0.39 | 1.24 ± 0.35 |  |
| Midline | 1.46 ± 0.46 | 1.10 ± 0.21 | -0.37 (-0.51, -0.22)** |
| Endline | 1.40 ± 0.43 | 1.13 ± 0.24 | -0.28 (-0.42, -0.13)** |
| **Mean anxiety sub-score** |  |  |  |
| Baseline | 1.20 ± 0.23 | 1.22 ± 0.28 |  |
| Midline | 1.33 ± 0.37 | 1.06 ± 0.12 | -0.28 (-0.40, -0.17)** |
| Endline | 1.25 ± 0.33 | 1.08 ± 0.16 | -0.18 (-0.33, -0.03)* |

^a^ Abbreviations used: CHW, community health worker; CCT, conditional cash transfer; HSCL, Hopkins Symptoms Checklist. Statistical significance: * p<0.05, ** p<0.01.

**Supplemental Table 6A** Modification of the effects of the CHW and CHW+CCT interventions on women’s Hopkin’s symptoms checklist-25 (HSCL-25) score, and depression and anxiety sub-scores at midline (9 months)^a^

|  | **HSCL-25 score** | | **Depression sub-score** | | **Anxiety sub-score** | |
| --- | --- | --- | --- | --- | --- | --- |
|  | CHW vs. Control  Mean difference  (95% CI) | CHW+CCT vs. Control  Mean difference  (95% CI) | CHW vs. Control  Mean difference  (95% CI) | CHW+CCT vs. Control  Mean difference  (95% CI) | CHW vs. Control  Mean difference  (95% CI) | CHW+CCT vs. Control  Mean difference  (95% CI) |
| Pregnancy status at trial enrollment |  |  |  |  |  |  |
| Not pregnant | -0.35 (-0.47, -0.23) | -0.29 (-0.41, -0.17) | -0.38 (-0.51, -0.24) | -0.30 (-0.44, -0.17) | -0.32 (-0.44, -0.19) | -0.27 (-0.39, -0.14) |
| Pregnant | -0.35 (-0.49, -0.21) | -0.35 (-0.48, -0.21) | -0.40 (-0.56, -0.24) | -0.41 (-0.57, -0.26) | -0.29 (-0.43, -0.14) | -0.25 (-0.39, -0.12) |
| p-value for interaction | 0.985 | 0.262 | 0.790 | 0.105 | 0.587 | 0.777 |
| Maternal education |  |  |  |  |  |  |
| Less than secondary | -0.38 (-0.49, -0.28) | -0.33 (-0.44, -0.23) | -0.42 (-0.54, -0.30) | -0.37 (-0.49, -0.25) | -0.33 (-0.44, -0.22) | -0.28 (-0.40, -0.17) |
| Secondary or higher | -0.18 (-0.38, 0.01) | -0.16 (-0.32, 0.00) | -0.20 (-0.44, 0.03) | -0.17 (-0.36, 0.02) | -0.15 (-0.34, 0.03) | -0.14 (-0.30, 0.02) |
| p-value for interaction | 0.029 | 0.015 | 0.060 | 0.029 | 0.039 | 0.034 |
| Marital status |  |  |  |  |  |  |
| Not married or cohabitating | -0.36 (-0.53, -0.19) | -0.24 (-0.39, -0.08) | -0.41 (-0.61, -0.21) | -0.23 (-0.42, -0.05) | -0.28 (-0.45, -0.12) | -0.25 (-0.40, -0.09) |
| Married or cohabitating | -0.35 (-0.47, -0.24) | -0.32 (-0.44, -0.20) | -0.38 (-0.51, -0.25) | -0.36 (-0.49, -0.23) | -0.31 (-0.43, -0.19) | -0.27 (-0.39, -0.15) |
| p-value for interaction | 0.931 | 0.212 | 0.753 | 0.133 | 0.695 | 0.720 |
| Parity |  |  |  |  |  |  |
| Nulliparous | -0.24 (-0.44, -0.05) | -0.23 (-0.41, -0.05) | -0.26 (-0.48, -0.03) | -0.24 (-0.45, -0.03) | -0.22 (-0.41, -0.04) | -0.21 (-0.39, -0.04) |
| Multiparous | -0.38 (-0.50, -0.25) | -0.33 (-0.46, -0.20) | -0.41 (-0.55, -0.27) | -0.36 (-0.50, -0.23) | -0.33 (-0.46, -0.19) | -0.28 (-0.41, -0.15) |
| p-value for interaction | 0.106 | 0.194 | 0.135 | 0.209 | 0.185 | 0.334 |
| Depression at baseline |  |  |  |  |  |  |
| No | -0.34 (-0.47, -0.21) | -0.33 (-0.46, -0.19) | -0.37 (-0.52, -0.22) | -0.36 (-0.51, -0.20) | -0.30 (-0.43, -0.17) | -0.28 (-0.42, -0.15) |
| Yes (HSCL-8 ≥1.06) | -0.37 (-0.50, -0.24) | -0.30 (-0.42, -0.18) | -0.40 (-0.55, -0.26) | -0.33 (-0.47, -0.19) | -0.31 (-0.44, -0.18) | -0.25 (-0.38, -0.13) |
| p-value for interaction | 0.625 | 0.648 | 0.636 | 0.721 | 0.809 | 0.573 |
| Depression at baseline |  |  |  |  |  |  |
| No | -0.33 (-0.47, -0.20) | -0.30 (-0.46, -0.14) | -0.32 (-0.49, -0.16) | -0.28 (-0.47, -0.09) | -0.35 (-0.49, -0.21) | -0.33 (-0.48, -0.18) |
| Yes (HSCL-25 ≥1.75) | -0.37 (-0.49, -0.24) | -0.31 (-0.43, -0.19) | -0.42 (-0.56, -0.28) | -0.36 (-0.50, -0.22) | -0.29 (-0.41, -0.16) | -0.24 (-0.36, -0.12) |
| p-value for interaction | 0.560 | 0.869 | 0.195 | 0.363 | 0.251 | 0.141 |

^a^ Abbreviations used: CHW, community health worker; CCT, conditional cash transfer; HSCL, Hopkins Symptoms Checklist.

**Supplemental Table 6B** Modification of the effects of the CHW and CHW+CCT interventions on women’s Hopkin’s symptoms checklist-25 (HSCL-25) score, and depression and anxiety sub-scores at endline (18 months)^a^

|  | **HSCL-25 score** | | **Depression sub-score** | | **Anxiety sub-score** | |
| --- | --- | --- | --- | --- | --- | --- |
|  | CHW vs. Control  Mean difference  (95% CI) | CHW+CCT vs. Control  Mean difference  (95% CI) | CHW vs. Control  Mean difference  (95% CI) | CHW+CCT vs. Control  Mean difference  (95% CI) | CHW vs. Control  Mean difference  (95% CI) | CHW+CCT vs. Control  Mean difference  (95% CI) |
| Pregnancy status at trial enrollment |  |  |  |  |  |  |
| Not pregnant | -0.27 (-0.40, -0.14) | -0.18 (-0.30, -0.05) | -0.32 (-0.45, -0.19) | -0.21 (-0.34, -0.08) | -0.19 (-0.33, -0.05) | -0.13 (-0.27, 0.01) |
| Pregnant | -0.32 (-0.48, -0.18) | -0.20 (-0.35, -0.06) | -0.36 (-0.51, -0.20) | -0.22 (-0.37, -0.07) | -0.27 (-0.42, -0.12) | -0.18 (-0.33, -0.03) |
| p-value for interaction | 0.326 | 0.556 | 0.609 | 0.846 | 0.111 | 0.256 |
| Maternal education |  |  |  |  |  |  |
| Less than secondary | -0.29 (-0.41, -0.16) | -0.19 (-0.31, -0.06) | -0.33 (-0.45, -0.21) | -0.22 (-0.34, -0.09) | -0.22 (-0.35, -0.08) | -0.15 (-0.28, -0.01) |
| Secondary or higher | -0.30 (-0.50, -0.11) | -0.16 (-0.33, 0.01) | -0.35 (-0.58, -0.12) | -0.17 (-0.37, 0.02) | -0.22 (-0.41, -0.03) | -0.12 (-0.29, 0.04) |
| p-value for interaction | 0.839 | 0.655 | 0.865 | 0.624 | 0.988 | 0.681 |
| Marital status |  |  |  |  |  |  |
| Not married or cohabitating | -0.18 (-0.34, -0.02) | -0.08 (-0.23, 0.08) | -0.18 (-0.35, 0.00) | -0.04 (-0.21, 0.13) | -0.18 (-0.35, -0.02) | -0.13 (-0.29, 0.03) |
| Married or cohabitating | -0.31 (-0.43, -0.19) | -0.21 (-0.33, -0.09) | -0.37 (-0.49, -0.25) | -0.26 (-0.37, -0.14) | -0.22 (-0.36, -0.09) | -0.15 (-0.28, -0.01) |
| p-value for interaction | 0.047 | 0.028 | 0.021 | 0.006 | 0.489 | 0.737 |
| Parity |  |  |  |  |  |  |
| Nulliparous | -0.21 (-0.40, -0.01) | -0.16 (-0.34, 0.02) | -0.24 (-0.46, -0.02) | -0.20 (-0.40, 0.00) | -0.18 (-0.37, 0.01) | -0.10 (-0.28, 0.08) |
| Multiparous | -0.31 (-0.44, -0.17) | -0.21 (-0.34, 0.07) | -0.36 (-0.49, -0.23) | -0.23 (-0.37, -0.10) | -0.23 (-0.37, -0.09) | -0.16 (-0.30, -0.02) |
| p-value for interaction | 0.225 | 0.505 | 0.231 | 0.719 | 0.504 | 0.345 |
| Depression at baseline |  |  |  |  |  |  |
| No | -0.33 (-0.46, -0.19) | -0.27 (-0.41, -0.14) | -0.40 (-0.53, -0.27) | -0.35 (-0.49, -0.21) | -0.21 (-0.36, -0.07) | -0.16 (-0.30, -0.01) |
| Yes (HSCL-8 ≥1.06) | -0.25 (-0.38, -0.12) | -0.13 (-0.25, -0.00) | -0.27 (-0.39, -0.14) | -0.12 (-0.24, 0.01) | -0.22 (-0.36, -0.07) | -0.14 (-0.28, 0.00) |
| p-value for interaction | 0.125 | 0.006 | 0.037 | <0.001 | 0.958 | 0.651 |
| Depression at baseline |  |  |  |  |  |  |
| No | -0.35 (-0.49, -0.21) | -0.29 (-0.45, -0.14) | -0.43 (-0.57, -0.28) | -0.36 (-0.53, -0.19) | -0.24 (-0.39, -0.09) | -0.20 (-0.36, -0.04) |
| Yes (HSCL-25 ≥1.75) | -0.26 (-0.39, -0.14) | -0.15 (-0.27, -0.03) | -0.30 (-0.42, -0.17) | -0.16 (-0.28, -0.05) | -0.21 (-0.35, -0.07) | -0.13 (-0.26, 0.01) |
| p-value for interaction | 0.116 | 0.024 | 0.065 | 0.014 | 0.534 | 0.221 |

^a^ Abbreviations used: CHW, community health worker; CCT, conditional cash transfer; HSCL, Hopkins Symptoms Checklist. Statistical significance: * p<0.05, ** p<0.01.

**Supplemental Table 7** Effects of the CHW and CHW+CCT interventions on women’s Hopkin’s symptoms checklist-25 (HSCL-25) score, and depression and anxiety sub-scores by percentage of completed CHW visits at endline (18 months)^a^

|  | CHW ≥90% Visits Completed vs. Control  Mean difference  (95% CI) | CHW <90% Visits Completed vs. Control  Mean difference  (95% CI) | CHW+CCT ≥90% Visits Completed vs. Control  Mean difference  (95% CI) | CHW+CCT <90% Visits Completed vs. Control  Mean difference  (95% CI) |
| --- | --- | --- | --- | --- |
| Participants (N) | 135 | 65 | 149 | 51 |
| Mean HSCL-25 score | -0.29 (-0.42, -0.17) | -0.27 (-0.40, -0.13) | -0.19 (-0.31, -0.06) | -0.17 (-0.31, -0.03) |
| Mean depression sub-score | -0.34 (-0.46, -0.21) | -0.31 (-0.45, -0.18) | -0.22 (-0.34, -0.10) | -0.17 (-0.32, -0.03) |
| Mean anxiety sub-score | -0.22 (-0.36, -0.09) | -0.20 (-0.34, -0.05) | -0.14 (-0.28, -0.00) | -0.17 (-0.31, -0.02) |

^a^ Abbreviations used: CHW, community health worker; CCT, conditional cash transfer; HSCL, Hopkins Symptoms Checklist.
